# Association of Perinatal and Childhood Ischemic Stroke With Attention-Deficit/Hyperactivity Disorder

**DOI:** 10.1001/jamanetworkopen.2022.8884

**Published:** 2022-04-26

**Authors:** Jenny Bolk, Eleni Simatou, Jonas Söderling, Lisa B. Thorell, Martina Persson, Heléne Sundelin

**Affiliations:** 1 Clinical Epidemiology Division, Department of Medicine Solna, Karolinska Institutet, Stockholm, Sweden; 2 Department of Clinical Science and Education, Södersjukhuset, Stockholm, Sweden; 3 Sachs’ Children and Youth Hospital, Södersjukhuset, Stockholm, Sweden; 4 Neuropediatric Unit, Department of Women’s and Children’s Health, Karolinska University Hospital, Karolinska Institutet, Stockholm, Sweden; 5 Department of Medical Epidemiology and Biostatistics, Karolinska Institutet, Stockholm, Sweden; 6 Department of Clinical Neuroscience, Karolinska Institutet, Stockholm, Sweden; 7 Division of Children’s and Women’s Health, Department of Biomedical and Clinical Sciences, Linköping University, Linköping, Sweden

## Abstract

**Importance:**

Early detection of attention-deficit/hyperactivity disorder (ADHD) plays a crucial role in reducing negative effects on everyday life, including academic failure and poor social functioning. Children who survive ischemic strokes risk major disabilities, but their risk of ADHD has not been studied in nationwide cohorts.

**Objective:**

To assess the risk of ADHD in children after pediatric ischemic stroke.

**Design, Setting, and Participants:**

Participants in this Swedish nationwide cohort study included 1320 children diagnosed with ischemic stroke recorded in linked Swedish national registers from January 1, 1969, to December 31, 2016, without prior ADHD diagnosis. Ten matched controls were identified for each index case, and first-degree relatives were identified for index individuals and controls. Analyses were stratified by perinatal and childhood strokes and presence of comorbid adverse motor outcomes and/or epilepsy. End of follow-up was the date of ADHD diagnosis, death, or December 31, 2016, whichever occurred first. Data analyses were performed August 1 to 28, 2021.

**Exposures:**

Pediatric ischemic stroke.

**Main Outcomes and Measures:**

Attention-deficit/hyperactivity disorder identified using codes from the *International Classification of Diseases, Ninth Revision*, and *International Statistical Classification of Diseases and Related Health Problems, Tenth Revision*, and/or prescribed ADHD medication recorded in the Medical Birth Register, National Patient Register, or Prescribed Drug Register after stroke. Cox proportional hazards regression was used to assess adjusted hazard ratios (aHRs) for ADHD after pediatric stroke, adjusting for parental age and ADHD in first-degree relatives.

**Results:**

Of 1320 children with stroke included in the analysis (701 boys [53.1%]), 75 (45 boys [60.0%]) were diagnosed with ADHD after stroke compared with 376 (252 boys [67.0%]) among the controls (aHR, 2.00 [95% CI, 1.54-2.60]). The risk was increased after both perinatal (aHR, 2.75 [95% CI ,1.65-4.60]) and childhood (aHR, 1.82 [95% CI, 1.34-2.48]) strokes and were similar if children born preterm or small for gestational age were excluded. Compared with controls, risks of ADHD were higher among children with perinatal stroke and adverse motor outcomes and/or epilepsy (aHR, 6.17 [95% CI, 2.80-13.62]) than among those without these comorbidities (aHR, 1.65 [95% CI, 0.80-3.42]). However, findings were similar in childhood stroke for children with adverse motor outcomes and/or epilepsy (aHR, 1.80 [95% CI, 1.12-2.89]) and among those without these comorbidities (aHR, 1.92 [95% CI, 1.28-2.90]).

**Conclusions and Relevance:**

This cohort study of 1320 children with pediatric ischemic stroke suggests that there is an increased risk of ADHD, particularly in children with adverse motor outcomes and/or epilepsy, compared with controls. The risk increases after childhood strokes regardless of comorbidities.

## Key Points

**Question:**

Does the risk of attention-deficit/hyperactivity disorder (ADHD) increase after pediatric stroke, and is that risk associated with family history of ADHD, adverse motor outcomes, or comorbid epilepsy?

**Findings:**

This nationwide cohort study of 1320 patients who had perinatal or childhood stroke and 13 141 matched controls found a 2-fold increased risk of ADHD after stroke. Development of adverse motor outcomes and/or epilepsy were associated with a further elevated risk in children with perinatal stroke.

**Meaning:**

These findings suggest that children may face an increased risk of ADHD after stroke and that surveillance for ADHD should be considered by pediatricians performing follow-up of children with pediatric stroke.

## Teaser

This cohort study uses data from nationwide Swedish registers to evaluate the risk of attention-deficit/hyperactivity disorder after perinatal and childhood ischemic stroke in a large cohort of children.

## Introduction

Children who survive an ischemic stroke face higher risks of neurological disabilities, including epilepsy, and adverse motor outcomes such as hemiplegia and cerebral palsy.^1,2,3,4,5,6,7^ Pediatric stroke may also increase the risk of attention-deficit/hyperactivity disorder (ADHD). The reported prevalence of ADHD after pediatric stroke has ranged from 13% to 50%.^8,9,10,11^ However, it is unclear whether ADHD risk is increased in children with ischemic stroke who do not have these comorbidities.

Symptoms of inattention and hyperactivity, as well as cognitive deficits in individuals with ADHD, often have a negative impact on academic achievements^12^ and social functioning.^13^ The prevalence of childhood ADHD in the general population is estimated to be approximately 3% to 5%.^13,14^ Although the exact etiology of ADHD is often unclear, there is a strong hereditable component,^15^ and associations have been found with low birth weight, prematurity,^16^ male sex,^17^ traumatic brain injuries, and brain tumors.^18,19^

It has been reported that 19% to 35% of children with cerebral palsy also have ADHD,^20^ which is approximately 6 times higher than in the general population. Children with epilepsy also have a 2.5 to 5.5 times higher risk of ADHD.^21^ Numerous studies have reported that cerebral palsy and epilepsy are common comorbidities after pediatric stroke,^1,2,3,4,5,6,7,22^ so we would also expect to see an increased risk of ADHD. However, it is unclear whether the risk of ADHD is increased in children with ischemic stroke who do not have these comorbidities. Previous studies assessing ADHD after pediatric stroke^8,9,10,11^ have had limited sample sizes and used varying definitions of ADHD. They have also been hospital based and/or did not include general population controls, which restricted the generalizability of their results.

The main aim of our study was to address these limitations by using data from nationwide registers to evaluate the risk of ADHD after pediatric ischemic stroke in a large cohort of children. We included family history of ADHD, prematurity, small for gestational age (SGA), comorbid adverse motor outcomes, and epilepsy in our analysis. We hypothesized that children who had pediatric stroke face a higher risk of ADHD even if not born preterm or SGA or not having comorbid adverse motor outcomes and/or epilepsy. Our secondary aim was to explore whether ADHD and ischemic stroke have a shared heredity. Because diverse conditions affecting the brain have been reported to be genetically related,^23,24,25^ we hypothesized that first-degree relatives of patients who had pediatric stroke would have an increased risk of ADHD. We explored this hypothesis by assessing the risk of ADHD in first-degree relatives of patients with pediatric stroke without ADHD.

## Methods

### Study Design and Study Population

This Swedish nationwide cohort study included children who were younger than 18 years with ischemic stroke between January 1, 1969, and December 31, 2016, and were alive 1 week after their stroke. They were identified using the National Patient Register^26,27^ and the Medical Birth Register.^28^ Individuals with ADHD before their stroke were excluded. The Total Population Register^26^ was used to identify 10 controls for each patient with stroke who were matched for sex, year of birth, and county of residence at the time of diagnosis. Parents, siblings, and offspring of patients and controls were identified from the Multigeneration Register, which is part of the Total Population Register. This cohort is part of PedStroke, a Swedish national study on pediatric stroke. Previous publications report mortality,^29^ risk of epilepsy,^22^ and adverse motor outcomes^30^ in the cohort. The study was approved by the regional ethics committee in Linköping. The authors had access to pseudonymized and encrypted data from Statistics Sweden only, therefore the ethics committee did not require individual informed consent.^31^ This report followed the Strengthening the Reporting of Observational Studies in Epidemiology (STROBE) and the Reporting of Studies Conducted Using Observational Routinely Collected Health Data (RECORD) reporting guidelines.

### Assessment Methods

Exposure consisted of ischemic stroke at younger than 18 years, defined by codes from the *International Classification of Diseases, Eighth Revision* (*ICD-8*), *International Classification of Diseases, Ninth Revision* (*ICD-9*), and *International Statistical Classification of Diseases and Related Health Problems, Tenth Revision* (*ICD-10*) (eTable 1 in the Supplement). Outcome consisted of a register entry of ADHD after the stroke diagnosis, defined by the relevant *ICD-9* or *ICD-10* code and/or prescription of ADHD medication documented in the Prescribed Drug Register^32^ (eTable 1 in the Supplement).

### Variables Used for Adjusted and Stratified Analyses

Perinatal stroke was defined as a diagnosis of an ischemic stroke 28 days after birth or earlier; childhood strokes were diagnosed after 28 days but before 18 years of age. Preterm birth was defined as birth before 37 plus 0 gestational weeks, and SGA was defined as birth weight of less than −2 SD for the mean birth weight for gestational age and sex according to Swedish reference data.^33^

Adverse motor outcomes consisted of a diagnosis of cerebral palsy, hemiparesis, tetraparesis, paraplegia, or any other paresis. These, together with epilepsy, were based on the *ICD-8*, *ICD-9*, and *ICD-10* codes in the National Patient Register (eTable 1 in the Supplement).

### Data Sources

The data from the Swedish national registers were linked using the unique personal registration number assigned to all residents at birth or immigration.^34^ The National Patient Register^27^ is a validated register^26^ that provides diagnostic information on Swedish residents since 1964, including all inpatient hospital care since 1987 and outpatient care since 2001. The Medical Birth Register^28^ prospectively collects information on antenatal and perinatal factors and neonatal diagnoses and covers more than 98% of Swedish births. The Prescribed Drug Register^32^ provides information on all prescribed drugs dispensed by Swedish pharmacies since 2005. The Multi-generation Register^35,36^ and the Total Population Register have almost complete coverage in individuals born from 1961 onward. Owing to the high coverage of the registers, all individuals in Sweden except very few cases are included when linking data from these registers.

The validity of the diagnoses of pediatric ischemic stroke in the National Patient Register and Medical Birth Register has been evaluated to be high. Review of medical records of parts of our cohort resulted in an overall positive predicted value of 89% for pediatric ischemic stroke, 96% for perinatal ischemic stroke, and 84% for childhood ischemic stroke.^37^ Diagnoses of ADHD in the National Patient Register and Prescribed Drug Register have also shown high validity.^38^ The registers were accessed on December 31, 2016.

### Statistical Analysis

Follow-up started at the date of stroke diagnosis and the corresponding date for the controls and ended at the first ADHD diagnosis, death, or December 31, 2016, whichever came first. The follow-up for first-degree relatives started at birth and ended as described above. When stratifying by comorbidity, follow-up started at the date of the first diagnosis of adverse motor outcomes and/or epilepsy.

Cox proportional hazards regression was used to calculate hazard ratios (HRs) and assess the risk of ADHD after stroke. Each index individual was compared with his or her matched controls using an internal stratifying model. Analyses were adjusted for parental age at the birth of the child and for any ADHD diagnosis of parents and siblings. Unadjusted results can be found in eTables 2 and 3 in the Supplement. Separate analyses were performed for perinatal strokes and childhood strokes because these conditions have somewhat separate clinical symptoms and treatments.

Sensitivity analyses were performed separately for children not born preterm or SGA, for sex, and for children with and without adverse motor outcomes and/or epilepsy because all these conditions have been reported to increase the risk of ADHD.^15,16,17^ We performed sensitivity analyses for individuals without adverse motor outcomes or epilepsy by censoring at the time of the first diagnosis of adverse motor outcomes or epilepsy after stroke. We also evaluated the risk of ADHD in parents, siblings, and offspring of children with stroke and controls.

The data analyses were performed from August 1 to 28, 2021, using SAS statistical software, version 9.4 (SAS Institute Inc), and Stata, version 16.0 (StataCorp LLC). A 95% CI that excluded 1.00 defined statistical significance.

## Results

### Study Population and Baseline Characteristics

After excluding 7 children with ADHD before their stroke, 1320 individuals who were younger than 18 years and had pediatric stroke between 1969 and 2016 (343 with perinatal stroke and 977 with childhood stroke) (Figure) were included in the analysis (701 boys [53.1%] and 619 girls [46.9%]). Baseline characteristics are presented in Table 1. Of the 1320 children, 75 (45 boys [60.0%] and 30 girls [40.0%]) were diagnosed with ADHD after stroke compared with 376 (252 boys [67.0%] and 124 girls [33.0%]) among the controls (adjusted HR [aHR], 2.00 [95% CI, 1.54-2.60]) (Table 2).

**Figure.**
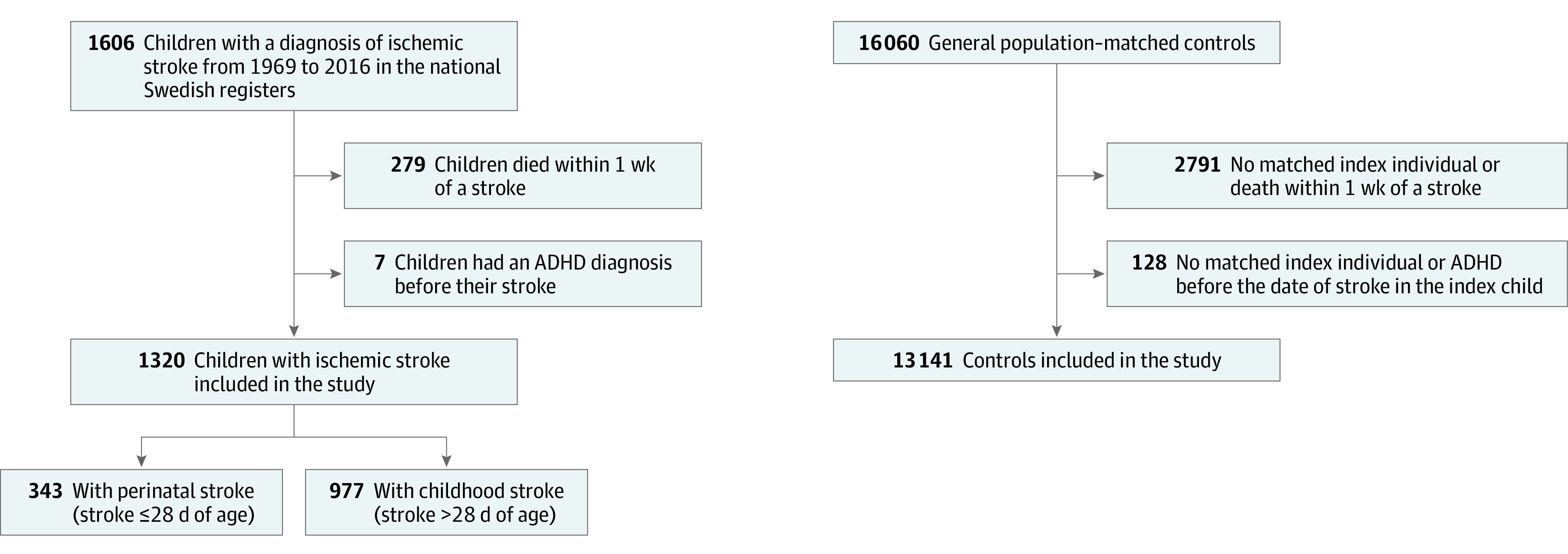
Flowchart of Study Population ADHD indicates attention-deficit/hyperactivity disorder.

**Table 1.** Baseline Characteristics for Individuals Who Had a Pediatric Ischemic Stroke and Matched Controls

Characteristic	Cohort group^a^	
	Children with pediatric ischemic stroke (n = 1320)	Matched controls (n = 13 141)
Demographic variables		
Sex		
Boys	701 (53.1)	6970 (53.0)
Girls	619 (46.9)	6171 (46.9)
Age at first stroke diagnosis		
Perinatal stroke (≤28 d)	343 (26.0)	3429 (26.1)
Childhood stroke (>28 d)	977 (74.0)	9712 (73.9)
Year of first stroke diagnosis		
1969-1986	136 (10.3)	1360 (10.3)
1987-1996	313 (23.7)	3129 (23.8)
1997-2005	253 (19.2)	2527 (19.2)
2006-2016	618 (46.8)	6125 (46.6)
Perinatal variables		
Small for gestational age	78 (5.9)	347 (2.6)
Missing birth weight	178 (13.5)	1612 (12.3)
Born preterm (<37 plus 0 wk)	123 (9.3)	671 (5.1)
Missing gestational age	165 (12.5)	1592 (12.1)
Comorbidity		
Adverse motor outcome^b^	424 (32.1)	43 (0.3)
Epilepsy^c^	303 (22.9)	120 (0.9)
Maternal age at birth of child		
Median (IQR), y	29.2 (25.5-33.1)	29.2 (25.4-33.1)
Missing data	7 (0.5)	108 (0.8)
Paternal age at birth of child		
Median (IQR), y	31.9 (27.8-36.0)	31.7 (27.8-36.0)
Missing data	29 (2.2)	301 (2.3)

^a^ Indicates after exclusion criteria were applied: individuals who died within the first week after a pediatric ischemic stroke or had a diagnosis of attention-deficit/hyperactivity disorder within 1 week after their stroke, along with the data from their matched controls. Unless otherwise indicated, data are expressed as number (%) of patients or controls.

^b^ Adverse motor outcome was defined as *International Classification of Diseases, Eighth Revision* (*ICD-8*), codes 343 and 344 (except 344.B, 344.C, and 344.G), *International Classification of Diseases, Ninth Revision* (*ICD-9*), codes 343, 342, and 344 (except 344.01 and 344.03), and *International Statistical Classification of Diseases and Related Health Problems, Tenth Revision* (*ICD-10*), codes (G80, G81, G82, and G83, except G83.0 and G83.4-6).

^c^ Epilepsy was defined as *ICD-8* code 345 (except 345.2), *ICD-9* code 345 (except 345Q), and *ICD-10* code G40.

**Table 2.** Risk of ADHD After Pediatric Ischemic Stroke^a^

Characteristic	Cohort group, No. (%)		Diagnosis of ADHD, No. (%)		Incidence rate per 1000 person-years, HR (95% CI)		Adjusted HR (95% CI)^b^
	Children with pediatric stroke	Matched controls	Children with pediatric stroke	Matched controls	Children with pediatric stroke	Matched controls	
Overall	1320 (100)	13 141 (100)	75 (5.7)	376 (2.9)	4.2 (3.3-5.2)	2.0 (1.8-2.2)	2.00 (1.54-2.60)
Sex							
Boys	701 (53.1)	6970 (53.0)	45 (6.4)	252 (3.6)	4.7 (3.3-6.1)	2.4 (2.1-2.7)	1.78 (1.27-2.49)
Girls	619 (46.9)	6171 (46.9)	30 (4.8)	124 (2.0)	3.7 (2.4-5.0)	1.4 (1.2-1.7)	2.45 (1.61-3.72)
Age at first stroke diagnosis							
Perinatal	343 (26.0)	3429 (26.1)	21 (6.1)	76 (2.2)	5.6 (3.2-8.0)	1.9 (1.5-2.3)	2.75 (1.65-4.60)
Childhood	977 (74.0)	9712 (73.9)	54 (5.5)	300 (3.1)	3.9 (2.8-4.9)	2.0 (1.8-2.2)	1.82 (1.34-2.48)
Year of first stroke diagnosis							
1986 or earlier	136 (10.3)	1360 (10.3)	1 (0.7)	25 (1.8)	0.2 (0.0-0.7)	0.5 (0.3-0.7)	0.38 (0.05-2.92)
1987-1996	313 (23.7)	3129 (23.8)	10 (3.2)	106 (3.4)	1.4 (0.5-2.3)	1.4 (1.1-1.7)	1.04 (0.53-2.02)
1997-2005	253 (19.2)	2527 (19.2)	30 (11.9)	138 (5.5)	9.1 (5.8-12.4)	3.8 (3.2-4.4)	2.12 (1.39-3.23)
2006-2016	618 (46.8)	6125 (46.6)	34 (5.5)	107 (1.7)	11.1 (7.4-14.8)	3.4 (2.7-4.0)	3.19 (2.10-4.86)

Abbreviations: ADHD, attention-deficit/hyperactivity disorder; HR, hazard ratio.

^a^ Attention-deficit/hyperactivity disorder was defined according to *International Classification of Diseases, Ninth Revision*, code 314 and *International Statistical Classification of Diseases and Related Health Problems, Tenth Revision*, code F90 and/or if the patient was in receipt of ADHD medication in the Prescribed Drug Register according to the Anatomical Therapeutic Chemical classification system codes N06BA01 to N06BA06, N06BA08 to N06BA12, and/or C02AC02 (guanfacine). Of the 75 index children with ADHD, 57 had been prescribed ADHD medication; of the 376 controls with ADHD, 324 had been prescribed ADHD medication. Individuals who died within the first week after a pediatric ischemic stroke or had a diagnosis of ADHD within 1 week after their stroke were excluded along with the data from their matched controls.

^b^ Conditioned on matching set (age, sex, year of birth, and county of residence at the time of stroke) and adjusted for maternal and paternal age at the birth of the child and any ADHD in parents or siblings.

### ADHD Risk After Pediatric Ischemic Stroke

The 1320 index individuals with pediatric stroke were twice as likely to have ADHD compared with controls when adjusting for parental age and a family history of ADHD (aHR, 2.00 [95% CI, 1.54-2.60]). The risk was similar between boys (aHR, 1.78 [95% CI, 1.27-2.49]) and girls (aHR, 2.45 [95% CI, 1.61-3.72]) (Table 2). This increased risk remained after excluding individuals born SGA and/or preterm (aHR, 2.19 [95% CI, 1.62-2.97]) (Table 3).

**Table 3.** Risk of ADHD After Pediatric Ischemic Stroke When Considering Perinatal Variables and Stroke Comorbidities^a^

Group	Cohort group, No. (%)		Diagnosis of ADHD, No. (%)		Incidence rate per 1000 person-years, HR (95% CI)		Adjusted HR (95% CI)^b^
	Children with pediatric stroke (n = 1320)	Matched controls (n = 13 141)	Children with pediatric stroke	Matched controls	Children with pediatric stroke	Matched controls	
**After excluding children who were born preterm (<37 plus 0 wk) and/or small for gestational age**							
Overall	967 (73.3)	8320 (63.3)	58 (6.0)	244 (2.9)	4.9 (3.7-6.2)	2.2 (2.0-2.5)	2.19 (1.62-2.97)
Perinatal stroke	283 (21.4)	2557 (19.5)	17 (6.0)	53 (2.1)	5.7 (3.0-8.4)	1.9 (1.4-2.4)	3.16 (1.78-5.61)
Childhood stroke	684 (51.8)	5763 (43.9)	41 (6.0)	191 (3.3)	4.7 (3.2-6.1)	2.4 (2.0-2.7)	1.94 (1.35-2.77)
**Stratification according to comorbidity, with follow-up from diagnosis of adverse motor outcomes or epilepsy**							
Children with adverse motor outcomes							
Overall	422 (32.0)	4183 (31.8)	28 (6.6)	120 (2.9)	6.4 (4.0-8.7)	2.4 (2.0-2.8)	2.10 (1.35-3.27)
Perinatal stroke	100 (7.6)	991 (7.5)	8 (8.0)	15 (1.5)	9.8 (3.0-16.5)	1.7 (0.9-2.6)	5.73 (2.34-14.03)
Childhood stroke	322 (24.4)	3192 (24.3)	20 (6.2)	105 (3.3)	5.6 (3.1-8.1)	2.5 (2.1-3.0)	1.60 (0.95-2.70)
Children with epilepsy							
Overall	293 (22.2)	2906 (22.1)	21 (7.2)	77 (2.6)	8.1 (4.6-11.5)	2.6 (2.0-3.1)	3.54 (2.13-5.90)
Perinatal stroke	80 (6.1)	796 (6.1)	8 (10.0)	15 (1.9)	12.4 (3.8-21.1)	2.2 (1.1-3.3)	5.69 (2.28-14.20)
Childhood stroke	213 (16.1)	2110 (16.1)	13 (6.1)	62 (2.9)	6.6 (3.0-10.3)	2.7 (2.0-3.3)	2.93 (1.56-5.48)
Children with adverse motor outcomes and/or epilepsy							
Overall	531 (40.2)	5255 (40.0)	35 (6.6)	145 (2.7)	6.3 (4.2-8.4)	2.3 (1.9-2.7)	2.37 (1.59-3.53)
Perinatal stroke	129 (9.8)	1277 (9.7)	11 (8.5)	20 (1.6)	10.0 (4.1-15.9)	1.7 (1.0-2.5)	6.17 (2.80-13.62)
Childhood stroke	402 (30.5)	3978 (30.3)	24 (6.0)	125 (3.1)	5.4 (3.2-7.6)	2.4 (2.0-2.9)	1.80 (1.12-2.89)
**Censoring at time of first diagnosis of adverse motor outcomes and/or epilepsy**							
Children with adverse motor outcomes were censored							
Overall	1320 (100)	13 141 (100)	47 (3.6)	374 (2.8)	3.5 (2.5-4.5)	2.0 (1.8-2.1)	1.95 (1.40-2.71)
Perinatal stroke	343 (26.0)	3429 (26.1)	13 (3.8)	76 (2.2)	4.4 (2.0-6.9)	1.9 (1.5-2.3)	1.99 (1.04-3.79)
Childhood stroke	977 (74.0)	9712 (73.9)	34 (3.5)	298 (3.1)	3.3 (2.2-4.3)	2.0 (1.7-2.2)	1.99 (1.35-2.93)
Children with epilepsy were censored							
Overall	1320 (100)	13 141 (100)	54 (4.1)	366 (2.8)	3.6 (2.6-4.5)	1.9 (1.7-2.1)	1.69 (1.24-2.31)
Perinatal stroke	343 (26.0)	3429 (26.1)	13 (3.8)	74 (2.2)	4.2 (1.9-6.5)	1.8 (1.4-2.3)	2.05 (1.08-3.92)
Childhood stroke	977 (74.0)	9712 (73.9)	41 (4.2)	292 (3.0)	3.4 (2.4-4.4)	1.9 (1.7-2.2)	1.62 (1.13-2.31)
Children with adverse motor outcomes and/or epilepsy were censored							
Overall	1320 (100)	13 141 (100)	40 (3.0)	364 (2.8)	3.3 (2.3-4.3)	1.9 (1.7-2.1)	1.81 (1.26-2.59)
Perinatal stroke	343 (26.0)	3429 (26.1)	10 (2.9)	74 (2.2)	3.8 (1.4-6.1)	1.8 (1.4-2.3)	1.65 (0.80-3.42)
Childhood stroke	977 (74.0)	9712 (73.9)	30 (3.1)	290 (3.0)	3.1 (2.0-4.3)	1.9 (1.7-2.1)	1.92 (1.28-2.90)

Abbreviations: ADHD, attention-deficit/hyperactivity disorder; HR, hazard ratio.

^a^ Attention-deficit/hyperactivity disorder was defined according to *International Classification of Diseases, Ninth Revision*, code 314 and *International Statistical Classification of Diseases and Related Health Problems, Tenth Revision*, code F90 and/or if the patient was in receipt of ADHD medication in the Prescribed Drug Register according to the Anatomical Therapeutic Chemical classification system codes N06BA01 to N06BA06, N06BA08 to N06BA12, and/or C02AC02 (guanfacine). Individuals who died within the first week after a pediatric ischemic stroke or had a diagnosis of ADHD within 1 week after their stroke were excluded along with the data from the matched controls.

^b^ Conditioned on matching set (age, sex, year of birth, and county of residence at the time of stroke) and adjusted for maternal and paternal age at the birth of the child and any ADHD in parents or siblings.

Compared with controls, the risk of developing ADHD was similar for the 422 children with adverse motor outcomes after stroke (aHR, 2.10 [95% CI, 1.35-3.27]) and among those without adverse motor outcomes (aHR, 1.95 [95% CI, 1.40-2.71]). The 293 children who had epilepsy after stroke had a slightly higher risk of ADHD compared with controls (aHR, 3.54 [95% CI, 2.13-5.90]) than among those who did not develop epilepsy (aHR, 1.69 [95% CI, 1.24-2.31]) (Table 3).

### Risk of ADHD After Perinatal Stroke

The 343 children with perinatal stroke also had an increased risk of ADHD (aHR, 2.75 [95% CI, 1.65-4.60]) (Table 2), which remained similar when excluding individuals born preterm or SGA (aHR, 3.16 [95% CI, 1.78-5.61]) (Table 3). The ADHD risk was elevated among all children with perinatal stroke and adverse motor outcomes and/or epilepsy (aHR, 6.17 [95% CI, 2.80-13.62]). The ADHD risk was also elevated compared with controls in the 100 patients with perinatal stroke who developed adverse motor outcome (aHR, 5.73 [95% CI, 2.34-14.03]) than among those without adverse motor outcomes (aHR, 1.99 [95% CI, 1.04-3.79]). In addition, the 80 patients with perinatal stroke and epilepsy had a higher risk compared with controls (aHR, 5.69 [95% CI, 2.28-14.20]) than among those without epilepsy (aHR, 2.05 [95% CI, 1.08-3.92]) (Table 3). When censoring the 129 children with adverse motor outcomes and/or epilepsy at time of the first diagnosis of adverse motor outcomes or epilepsy, the risk of ADHD was no longer increased in children with perinatal stroke (aHR, 1.65 [95% CI, 0.80-3.42]) (Table 3).

### Risk of ADHD After Childhood Stroke

The risk of ADHD after childhood stroke was increased (aHR, 1.82 [95% CI, 1.34-2.48]) (Table 2), and it remained similar when excluding children born preterm or SGA (aHR, 1.94 [95% CI, 1.35-2.77]) (Table 3). The risk for ADHD was elevated among all children with childhood stroke who had adverse motor outcomes and/or epilepsy (aHR, 1.80 [95% CI, 1.12-2.89]). The risk of ADHD compared with controls was similar for the 322 children with adverse motor outcomes (aHR, 1.60 [95% CI, 0.95-2.70]) than among those without adverse motor outcomes (aHR, 1.99 [95% CI, 1.35-2.93]). The risk of ADHD compared with controls in the 213 children with childhood stroke who had epilepsy was not significantly higher (aHR, 2.93 [95% CI, 1.56-5.48]) than among children without epilepsy (aHR, 1.62 [95% CI, 1.13-2.31]). After censoring the 402 individuals with comorbid adverse motor outcomes and/or epilepsy, the risk of ADHD was still increased (aHR, 1.92 [95% CI, 1.28-2.90]) (Table 3).

### Risk of ADHD in Relatives of Patients With Stroke 

The siblings of patients with stroke had a higher risk of ADHD than the siblings of controls (aHR, 1.38 [95% CI, 1.09-1.74]), and the same result was seen for both perinatal and childhood stroke (Table 4). When only including patients with pediatric stroke and without ADHD, the risk of ADHD in siblings was still elevated (aHR, 1.47 [95% CI, 1.14-1.90]).

**Table 4.** Risk of ADHD in Parents, Siblings, and Offspring of Individuals With Pediatric Ischemic Stroke

Included individuals	ADHD cases in relatives, No. (%)		HR (95% CI)	Adjusted HR (95% CI)^a^
	Children with pediatric ischemic stroke	Matched controls		
With or without ADHD				
Overall	160 (2.8)	1026 (2.0)	1.45 (1.23-1.72)	1.46 (1.23-1.73)
Siblings	100 (4.4)	594 (3.5)	1.37 (1.09-1.72)	1.38 (1.09-1.74)
Parents	46 (1.5)	342 (1.1)	1.34 (0.98-1.84)	1.30 (0.92-1.83)
Offspring	14 (4.6)	90 (4.1)	1.26 (0.67-2.37)	1.32 (0.70-2.50)
Perinatal stroke (aged ≤28 d)	37 (3.1)	228 (2.1)	1.54 (1.08-2.19)	1.54 (1.07-2.19)
Stroke in childhood (aged >28 d)	123 (2.7)	798 (2.0)	1.43 (1.18-1.73)	1.43 (1.18-1.74)
Without ADHD				
Overall	137 (2.5)	851 (1.7)	1.54 (1.28-1.85)	1.53 (1.27-1.84)
Siblings	85 (4.0)	500 (3.0)	1.48 (1.15-1.90)	1.47 (1.14-1.90)
Parents	38 (1.3)	269 (0.9)	1.50 (1.06-2.13)	1.42 (0.96-2.08)
Offspring	14 (4.7)	82 (3.8)	1.40 (0.74-2.65)	1.46 (0.77-2.78)

Abbreviations: ADHD, attention-deficit/hyperactivity disorder; HR, hazard ratio.

^a^ Adjusted for maternal and paternal age at the birth of the child.

## Discussion

This nationwide cohort study of 1320 patients with pediatric stroke identified a 2-fold increased risk of ADHD after pediatric ischemic stroke. That risk remained increased after known risk factors for ADHD were considered, such as a family history of ADHD,^15^ parental age,^39^ prematurity, or SGA.^16^ The increased risk was seen after both perinatal and childhood stroke, which is in line with the study by Williams et al.^10^ Other studies^9,11^ did not distinguish between perinatal and childhood strokes. Epilepsy and adverse motor outcomes have been associated with ADHD in the general population^21,40^ and are common sequelae after pediatric stroke.^41,42^ Therefore, we also ran sensitivity analyses that censored individuals with these comorbidities.

Previous studies have also reported increased risks of ADHD diagnosis or ADHD symptom level after pediatric strokes.^9,10,11,43^ However, most of these studies included small cohorts.^9,11,43^ The exception was the study by Williams et al,^10^ which included 275 patients with pediatric stroke. To our knowledge, this is also the only study except ours that accounted for family history of ADHD. To our knowledge, our study is the largest to date to report an increased risk of a clinical diagnosis of ADHD after pediatric stroke.

### Risk of ADHD After Perinatal Stroke

The risk of ADHD was increased after perinatal stroke and increased 6 times if the child also had adverse motor outcomes. The expected adverse motor outcome after perinatal stroke is cerebral palsy. Attention-deficit/hyperactivity disorder is more common in individuals with cerebral palsy than in the general population,^20^ and a Swedish population-based study^44^ reported that half of the 200 children with cerebral palsy had positive results for ADHD.

As expected, children diagnosed with comorbid epilepsy after perinatal stroke had a higher risk of ADHD compared with controls than those with perinatal stroke without epilepsy. This result echoed the findings of Williams et al^10^ and Auvin et al.^21^ Clinicians are already advised to screen for ADHD when children have epilepsy because this outcome is a well-known comorbid condition.^21^ Genetic and environmental factors can contribute to the comorbidity of ADHD in epilepsy, as well as an imbalance between excitation and inhibition in the brain.^45^ This imbalance could also contribute to the development of ADHD.^46^

### Risk of ADHD After Childhood Stroke

The risk of ADHD was also increased after childhood stroke, and the presence of adverse motor outcomes was not associated with the risk. Adverse motor outcomes after childhood stroke most commonly consist of hemiplegia and, for some of the younger children, cerebral palsy, in addition to less severe palsies and paresis that are often caused by a smaller insult to the brain.

Epilepsy seems to be a risk factor for ADHD after both childhood and perinatal strokes. However, compared with controls, the risk of ADHD was also increased after childhood stroke for those without adverse motor outcomes or epilepsy.

It has been reported that the risk of ADHD is increased after other brain insults during childhood, such as severe traumatic brain injuries.^18^ In these cases, a decrease in the organization of white matter seems to play a crucial role in the development of ADHD,^47^ and this decrease could also be important in other disorders. Whether this outcome is the case after pediatric stroke needs to be elucidated.

### Risk of ADHD in First-Degree Relatives of Patients With Stroke

We found an increased risk of ADHD in the siblings of patients with pediatric stroke with and without ADHD. The rationale behind evaluating ADHD in relatives of these patients is that previous research^23^ has demonstrated a high degree of genetic correlation between different psychiatric disorders. Thus, our results support the hypothesis that there is a genetic disposition that increases the risk of both ADHD and stroke.

### Strengths and Limitations

To our knowledge, this is the largest study of risk of ADHD in patients with pediatric stroke to date, and our results confirm findings of smaller, hospital-based studies.^9,10,11^ Pediatric stroke is a relatively rare event, and this factor resulted in low power in most of the previous studies. Another major strength of the present study is its nationwide design. As recommended for follow-up studies,^48^ we matched controls and compared risks of ADHD between patients with stroke and controls. All data were collected prospectively. We adjusted for important confounders, such as ADHD in first-degree relatives, parental age, prematurity, and SGA. A diagnosis of ADHD was identified based on clinical *ICD-9* and *ICD-10* codes in contrast to previous studies using diagnoses based on questionnaires or research tests,^11,43^ which may overestimate the prevalence of ADHD.^49^ Stratified analyses of the risks of perinatal or childhood strokes and neurological comorbidities were performed. Data were retrieved from registers with high validity. The stroke diagnosis in our cohort has been validated.

This study has some limitations. There is a possibility of detection bias by misclassification or underreporting, such as underreporting of stroke before the advent of modern imaging, and for surveillance bias if patients with stroke were more carefully observed. Some individuals might have had ADHD before the stroke, although the ADHD diagnosis was registered after the stroke. Fewer controls had ADHD (2.9%) than previously reported in Swedish national registers (4.3%),^50^ possibly because the follow-up time was limited to the date of the stroke diagnosis in the matched index case and that a diagnosis code for ADHD is only available after *ICD-9* was implemented in 1987. The number of individuals in the exposed group was relatively small for some analyses, with wide 95% CIs for some aHRs. If possible, our results should therefore be confirmed in other large cohorts of patients with pediatric stroke.

Owing to the limited number of individuals in some subgroups, we were also only able to report results compared with controls instead of making direct comparisons between children with and without comorbidities. We could not distinguish between different types of ischemic stroke, but a previous validation study^37^ showed that 90% were arterial ischemic strokes. The proportion of perinatal strokes in our cohort was lower than expected. Some neonatal units may have used unspecific *ICD* codes (eg, cerebral ischemia) for perinatal stroke cases. Our study population was probably heterogenous in terms of the localization and size of the strokes. Outpatient diagnoses of ADHD have only been available since 2001. However, we estimate risks compared with controls, and the risks are probably not as influenced by this factor as the prevalence rates. We did not evaluate other mental health outcomes, socioeconomic status, parental health, or other child health variables that could have influenced our findings. In addition, we cannot rule out unmeasured and residual confounding variables that may have influenced the result.

## Conclusions

The findings of this nationwide cohort study suggest that pediatric ischemic stroke was associated with a risk of ADHD irrespective of whether there was a family history of ADHD. This risk was even higher if there were adverse motor outcomes and/or epilepsy after perinatal stroke. Furthermore, the risk was increased in patients with childhood stroke who did not have these comorbidities. Ongoing surveillance for signs of ADHD should form an important part of follow-up programs after pediatric strokes, especially if individuals have comorbid adverse motor outcomes or epilepsy.

## Notes

Supplement. **eTable 1.** *International Classification of Diseases* (*ICD*) Codes and Anatomical Therapeutic Chemical Classification System Codes **eTable 2.** Risk of Attention-Deficit/Hyperactivity Disorder (ADHD) After Stroke, Unadjusted and Only Adjusted for Parental Age **eTable 3.** Stratified Analyses on Risk of Attention-Deficit/Hyperactivity Disorder (ADHD) After Stroke, Unadjusted and Only Adjusted for Parental Age
