# Supplementary material for: Association of Perinatal and Childhood Ischemic Stroke With Attention-Deficit/Hyperactivity Disorder
Source: JAMA Netw Open. 2022 Apr 26;5(4):e228884. doi: 10.1001/jamanetworkopen.2022.8884 (PMC9044107; doi:10.1001/jamanetworkopen.2022.8884)
Supplement: Supplement. — eTable 1. International Classification of Diseases (ICD) Codes and Anatomical Therapeutic Chemical Classification System Codes eTable 2. Risk of Attention-Deficit/Hyperactivity Disorder (ADHD) After Stroke, Unadjusted and Only Adjusted for Parental Age eTable 3. Stratified Analyses on Risk of Attention-Deficit/Hyperactivity Disorder (ADHD) After Stroke, Unadjusted and Only Adjusted for Parental Age [file jamanetwopen-e228884-s001.pdf]

Supplementary Online Content

Bolk J, Simatou E, Söderling J, Thorell LB, Persson M, Sundelin H. Association of perinatal and childhood ischemic stroke with attention-deficit/hyperactivity disorder. *JAMA Netw Open*. 2022;5(4):e228884.
doi:10.1001/jamanetworkopen.2022.8884

eTable 1. *International Classification of Diseases* (*ICD*) Codes and Anatomical Therapeutic Chemical Classification System Codes

eTable 2. Risk of Attention-Deficit/Hyperactivity Disorder (ADHD) After Stroke, Unadjusted and Only Adjusted for Parental Age

eTable 3. Stratified Analyses on Risk of Attention-Deficit/Hyperactivity Disorder (ADHD) After Stroke, Unadjusted and Only Adjusted for Parental Age

This supplementary material has been provided by the authors to give readers additional information about their work.

© 2022 Bolk J et al. *JAMA Network Open*.

eTable 1. ***International Classification of Diseases* (*ICD*) Codes and Anatomical Therapeutic Chemical Classification System Codes**

**Diagnoses**	**Codes**
Ischemic stroke	Defined by ICD codes: ICD-8 and ICD-9: 433, 434, 436; ICD-10: I63, I64
Attention-deficit/hyperactivity disorder (ADHD)	Defined by ICD codes ICD-9: 314; ICD-10: F90<br>and/or if the patient had been prescribed ADHD medication in the Prescribed Drug Register (according to the Anatomical Therapeutic Chemical classification system codes N06BA01-N06BA06, N06BA08-N06BA12 and/or C02AC02
Adverse motor outcome	Defined as a diagnosis of cerebral palsy, hemiparesis, tetraparesis/paraplegia or any other paresis more than one week after the stroke and based the ICD codes: ICD 8: 343, 344 (except for 344. B, C, G); ICD 9: 343, 342, 344 (except for 344.01, 344.03); ICD 10: G80, G81, G82, G83 (except for G83.0, G83.4-6).
Epilepsy	Defined by ICD codes ICD-8: 345 (except for 345.2); ICD-9: 345 (except for 345Q); ICD-10: G40.

© 2022 Bolk J et al. *JAMA Network Open*.

eTable 2. Risk of Attention-Deficit/Hyperactivity Disorder (ADHD) After Stroke, Unadjusted and Only Adjusted for Parental Age

Group		Pediatric ischemic stroke,		n events - a diagnosis of ADHD (%),		HR[b] (95% CI)	HR[c] (95% CI), adjusted for parental age
		Index children n (%)	Controls n (%)	Index children n (%)	Controls n (%)		
Overall		1 320 (100)	13 141 (100)	75 (5.7)	376 (2.9)	2.25 (1.75-2.89)	2.33 (1.81-3.00)
Sex	Male	701 (53.1)	6 970 (53.0)	45 (6.4)	252 (3.6)	2.02 (1.46-2.78)	2.09 (1.51-2.89)
	Female	619 (46.9)	6 171 (47.0)	30 (4.8)	124 (2.0)	2.70 (1.80-4.04)	2.81 (1.87-4.24)
Age at first stroke diagnosis	Perinatal ≤28 days	343 (26.0)	3 429 (26.1)	21 (6.1)	76 (2.2)	3.21 (1.96-5.26)	3.34 (2.02-5.52)
	Childhood >28 days	977 (74.0)	9 712 (73.9)	54 (5.5)	300 (3.1)	2.01 (1.50-2.69)	2.08 (1.55-2.80)
Year of first stroke diagnosis	≤1986	136 (10.3)	1 360 (10.3)	1 (0.7)	25 (1.8)	0.45 (0.06-3.37)	0.42 (0.06-3.16)
	1987 - 1996	313 (23.7)	3 129 (23.8)	10 (3.2)	106 (3.4)	1.08 (0.56-2.07)	1.17 (0.60-2.25)
	1997 - 2005	253 (19.2)	2 527 (19.2)	30 (11.9)	138 (5.5)	2.52 (1.69-3.77)	2.54 (1.70-3.81)
	2006 - 2016	618 (46.8)	6 125 (46.6)	34 (5.5)	107 (1.7)	3.39 (2.30-5.02)	3.68 (2.45-5.51)
Children followed up in the first 5 years after their stroke, by year of diagnosis	≤1986	136 (10.3)	1 360 (10.3)	0	0		
	1987 - 1996	313 (23.7)	3 129 (23.8)	0	0		
	1997 - 2005	253 (19.2)	2 527 (19.2)	6 (2.4)	13 (0.5)	4.97 (1.87-13.24)	4.95 (1.79-13.64)
	2006 - 2016	618 (46.8)	6 125 (46.6)	17 (2.8)	65 (1.1)	2.74 (1.60-4.70)	2.77 (1.58-4.88)

[a]*Individuals who died within the first week after a pediatric ischemic stroke or had a diagnosis of ADHD within one week after their stroke were excluded along with the data from the matched controls.*
[b]*Conditioned on matching set (age, sex, year of birth, and county of residence at the time of stroke).*
[c]*Conditioned on matching set and further adjusted for maternal and paternal age at the birth of the child.*
Attention-deficit/hyperactivity disorder (ADHD) was defined according to international classification of disease (ICD) codes (ICD-9 (314), ICD-10 (F90) and/or if the patient was in receipt of ADHD medication in the Prescribed Drug Register according to the Anatomical Therapeutic Chemical classification system codes N06BA01-N06BA06, N06BA08-N06BA12 and/or C02AC02 Guanfacin.

© 2022 Bolk J et al. *JAMA Network Open*.

eTable 3. Stratified Analyses on Risk of Attention-Deficit/Hyperactivity Disorder (ADHD) After Stroke, Unadjusted and Only Adjusted for Parental Age

Group		Pediatric ischemic stroke,		n events - a diagnosis of ADHD (%)		HR[a] (95% CI), unadjusted	HR[b] (95% CI), adjusted for maternal and paternal age
		Index children n (%)	Controls n (%)	Index children n (%)	Controls n (%)		
After excluding children who were born preterm (<37+0 weeks) and/or small for gestational age	Overall	967 (73.3)	8 320 (63.3)	58 (6.0)	244 (2.9)	2.37 (1.77-3.17)	2.42 (1.80-3.24)
	Perinatal stroke	283 (21.4)	2 557 (19.5)	17 (6.0)	53 (2.1)	3.37 (1.93-5.89)	3.79 (2.14-6.71)
	Childhood stroke	684 (51.8)	5 763 (43.9)	41 (6.0)	191 (3.3)	2.11 (1.50-2.97)	2.10 (1.48-2.96)
**Stratification according to comorbidity, with follow-up from diagnosis of adverse motor outcomes or epilepsy**							
Children with adverse motor outcomes	Overall	422 (32.0)	4 183 (31.8)	28 (6.6)	120 (2.9)	2.58 (1.70-3.92)	2.54 (1.67-3.87)
	Perinatal stroke	100 (7.6)	991 (7.5)	8 (8.0)	15 (1.5)	5.63 (2.36-13.43)	6.37 (2.56-15.87)
	Childhood stroke	322 (24.4)	3 192 (24.3)	20 (6.2)	105 (3.3)	2.12 (1.31-3.44)	2.05 (1.25-3.34)
Children with epilepsy	Overall	293 (22.2)	2 906 (22.1)	21 (7.2)	77 (2.6)	3.13 (1.91-5.12)	3.26 (1.98-5.35)
	Perinatal stroke	80 (6.1)	796 (6.1)	8 (10.0)	15 (1.9)	6.00 (2.49-14.48)	6.10 (2.48-14.97)
	Childhood stroke	213 (16.1)	2 110 (16.1)	13 (6.1)	62 (2.9)	2.42 (1.32-4.44)	2.53 (1.37-4.68)
Children with adverse motor outcomes or epilepsy	Overall	531 (40.2)	5 255 (40.0)	35 (6.6)	145 (2.8)	2.75 (1.89-4.01)	2.76 (1.89-4.03)
	Perinatal stroke	129 (9.8)	1 277 (9.7)	11 (8.5)	20 (1.6)	6.35 (2.97-13.56)	6.85 (3.13-15.00)
	Childhood stroke	402 (30.5)	3 978 (30.3)	24 (6.0)	125 (3.1)	2.19 (1.40-3.40)	2.18 (1.39-3.41)
**Censor at time of first diagnosis of adverse motor outcome and/or epilepsy**							
Children with adverse motor outcome were censored	Overall	1320(100)	13 141 (100)	47 (3.6)	374 (2.8)	2.10 (1.53-2.88)	2.24 (1.63-3.08)
	Perinatal stroke	343 (26.0)	3 429 (26.1)	13 (3.8)	76 (2.2)	2.53 (1.38-4.67)	2.58 (1.39-4.79)
	Childhood stroke	977 (74.0)	9 712 (73.9)	34 (3.5)	298 (3.1)	1.97 (1.36-2.85)	2.13 (1.46-3.09)
Children with epilepsy were censored	Overall	1 320 (100)	13 141 (100)	54 (4.1)	366 (2.8)	2.05 (1.53-2.75)	2.12 (1.58-2.86)
	Perinatal stroke	343 (26.0)	3 429 (26.1)	13 (3.8)	74 (2.2)	2.56 (1.39-4.73)	2.69 (1.45-5.01)
	Childhood stroke	977 (74.0)	9 712 (73.9)	41 (4.2)	292 (3.0)	1.93 (1.38-2.70)	1.99 (1.42-2.80)

© 2022 Bolk J et al. *JAMA Network Open*.

**Children with adverse motor outcomes/epilepsy were censored**	**Overall**	1 320(100)	13 141 (100)	40 (3.0)	364 (2.8)	1.99 (1.42-2.79)	2.10 (1.49-2.96)
	**Perinatal stroke**	343 (26.0)	3 429 (26.1)	10 (2.9)	74 (2.2)	2.14 (1.08-4.25)	2.24 (1.12-4.48)
	**Childhood stroke**	977 (74.0)	9 712 (73.9)	30 (3.1)	290 (3.0)	1.94 (1.31-2.88)	2.06 (1.39-3.07)

[a]Conditioned on matching set (age, sex, year of birth, and county of residence at the time of stroke).
[b]Conditioned on matching set and further adjusted for maternal and paternal age at the birth of the child.
Attention-deficit/hyperactivity disorder (ADHD) was defined according to international classification of disease (ICD) codes (ICD-9 (314), ICD-10 (F90) and/or if the patient was in receipt of ADHD medication in the Prescribed Drug Register according to the Anatomical Therapeutic Chemical classification system codes N06BA01-N06BA06, N06BA08-N06BA12 and/or C02AC02 Guanfacin.

© 2022 Bolk J et al. *JAMA Network Open*.
